# RACIAL/ETHNIC DISPARITIES IN HEALTH: THE ROLE OF PHYSICALLY DEMANDING WORK

**DOI:** 10.1093/geroni/igad104.0860

**Published:** 2023-12-21

**Authors:** Sung Park, Anne Pebley, Noreen Goldman, Borianna Pratt, Mara Sheftel

**Affiliations:** University of Massachusetts Boston, Boston, Massachusetts, United States; University of California, Los Angeles, Los Angeles, California, United States; Princeton University, Princeton, New Jersey, United States; Princeton University, Princeton, New Jersey, United States; Penn State University, State College, Pennsylvania, United States

## Abstract

Work is an important contributor to racial/ethnic health disparities. Because older-age functional limitations are associated with accumulated physical wear and tear, investigating work-related mechanisms that differentially expose Black and Hispanic Americans to difficult work conditions over time is an important step towards understanding these disparities. Using data on lifetime work histories, this study investigates the role of accumulated years of physically demanding work (PDW) in young and middle adulthood on the number of functional limitations at age 60 (FL60). It also assesses whether cumulative PDW accounts for the observed racial/ethnic differences in FL60 among U.S.-born Black, Hispanic, and White respondents. Results show cumulative PDW is strongly associated with FL60 and that it accounts for a part of the racial/ethnic gap in FL60 in the presence of extensive control variables. The findings illustrate the importance of work as a dynamic process that ultimately influences the health of a diverse, aging population. Policy implications are addressed.

